# Opt-out of Voluntary HIV Testing: A Singapore Hospital's Experience

**DOI:** 10.1371/journal.pone.0034663

**Published:** 2012-04-06

**Authors:** Arlene C. Chua, Yee Sin Leo, Philippe Cavailler, Christine Chu, Aloysius Ng, Oon Tek Ng, Prabha Krishnan

**Affiliations:** 1 Department of Infectious Disease, Tan Tock Seng Hospital, Singapore, Singapore; 2 Department of Epidemiology Research, Regional Emerging Diseases Intervention Centre, Singapore, Singapore; 3 Department of Laboratory Medicine, Tan Tock Seng Hospital, Singapore, Singapore; McGill University Health Centre - McGill University, Canada

## Abstract

**Introduction:**

Since 2008, the Singapore Ministry of Health (MOH) has expanded HIV testing by increasing anonymous HIV test sites, as well as issuing a directive to hospitals to offer routine voluntary opt out inpatient HIV testing. We reviewed this program implemented at the end of 2008 at Tan Tock Seng Hospital (TTSH), the second largest acute care general hospital in Singapore.

**Methods and Findings:**

From January 2009 to December 2010, all inpatients aged greater or equal than 21 years were screened for HIV unless they declined or were not eligible for screening. We reviewed the implementation of the Opt Out testing policy. There were a total of 93,211 admissions; 41,543 patients were included based on HIV screening program eligibility criteria. Among those included, 79% (n = 32,675) opted out of HIV screening. The overall acceptance rate was 21%. Majority of eligible patients who were tested (63%) were men. The mean age of tested patients was 52 years. The opt out rate was significantly higher among females (OR: 1.5, 95%CI: 1.4–1.6), aged >60 years (OR: 2.3, 95%CI: 2.2–2.4) and Chinese ethnicity (OR: 1.7, 95%CI:1.6–1.8). The false positive rate of the HIV screening test is 0.56%. The proportion of patients with HIV infection among those who underwent HIV screening is 0.18%. All16 confirmed HIV patients were linked to care.

**Conclusion:**

The default opt-in rate of inpatient HIV testing was low at Tan Tock Seng Hospital, Singapore. Efforts to address individual HIV risk perception and campaigns against HIV stigma are needed to encourage more individuals to be tested for HIV.

## Introduction

In 2010, the Singapore Ministry of Health (MOH) reported 441 new diagnoses of HIV infection, resulting in a cumulative total of 4,845 patients with HIV/AIDS in the country. More than half of patients newly diagnosed with HIV infection are in the advanced stage of the disease [1]. Prevalence rates of HIV infection range from 0.1% [1] to an estimated 0.3% [2]. From 2008 to the present, the MOH has expanded HIV testing by increasing the number of anonymous HIV test sites, making rapid HIV tests available, as well as issuing a directive to hospitals to offer routine voluntary screening with opt out inpatient HIV testing.

These recommendations followed more recent United States Centers for Disease Control guidelines on HIV screening [3], and the results of an unlinked, anonymous HIV seroprevalence survey conducted by the Singapore MOH in 2007 in 5 public hospitals which reported the prevalence of undiagnosed HIV infection at 0.28% [4]. These efforts to make HIV testing routine in medical settings have begun to achieve the dual public health goals of increasing the proportion of individuals aware of their status and providing these patients with access to HIV care and treatment as well as constituting an important prevention tool to slow the spread of HIV.

Tan Tock Seng Hospital (TTSH) is Singapore's second largest acute care general hospital, with 1,481 beds in a geographically central location within Singapore. Here we report the results of a voluntary inpatient opt out HIV testing program at TTSH, in compliance with the MOH directive for routine opt out HIV testing.

## Methods

From January 2009 to December 2010, designated HIV counselors from TTSH offered bedside educational information and HIV screening to inpatients admitted at TTSH if the medical team deemed the patient eligible for HIV screening. All inpatients aged 21 years and above were screened for HIV unless they decline or are not eligible for screening. Patients were not approached about HIV screening if they had dementia, delirium, were admitted to the intensive care unit or high dependency unit, had a do-not-resuscitate (DNR) order, had known HIV, or had had an HIV test within the previous 6 months.

All patients who agreed to HIV screening had blood drawn for an HIV antibody test using Electrochemiluminescence Immunoassay or ECLIA (Elecsys HIV Combi Test, Roche Diagnostics) done in the Department of Laboratory Medicine. This test is an immunoassay for the in vitro determination of HIV-1 p 24 antigen and antibodies to HIV-1, including group O, and HIV-2 in human serum and plasma. Any patient with a reactive HIV antibody test received a Western blot confirmation at the National HIV Reference Laboratory. It is mandatory in Singapore to send these samples to this laboratory for confirmation of HIV.

The combined cost of the HIV antibody and Western Blot test was between S$6.00 to S$10.00. Costs varied depending on level of government subsidy. The cost of the test without subsidy is S$22.26.

Non-reactive HIV screening test results were conveyed to the patients by the HIV counselors. While offering bedside educational information patients were advised that should their results not be available at the time of discharge, they could call a designated number to collect the result at a later date; this information is also provided in the educational brochure given to patients at the time of admission. Reactive HIV screening test results were conveyed to the primary physician who was directed to follow up on the confirmatory HIV Western blot assay result and was deemed responsible for informing patients. On confirmation with HIV Western blot assay, patients were referred by the primary care physician to an Infectious Disease physician in TTSH for specialty HIV care at the Communicable Disease Centre (CDC).

From the 24^th^ to 30^th^ of November 2010, all patients who opted out of the HIV screening test were asked about their reasons for opting out. The reasons were collected and recorded with their verbal consent without other identifiers recorded.

We evaluated the total number of patients who were admitted in TTSH during the 2-year review period and determined the number of patients who agreed to be HIV tested, number of patients with reactive screening tests, number of confirmed HIV infections among those tested and the rate of linkages to HIV care and treatment. Data was obtained through the Management Information Department of the Office of Clinical Governance of TTSH. We obtained demographic data on sex, age, ethnicity, and International Classification of Disease (ICD) diagnosis on patients who met eligibility criteria. Data from patients who opted out were compared to those who opted in for HIV screening test. SPSS for Windows Version 18.0 was used for all statistical analyses. We compared proportions using Pearson Chi-square, means using ANOVA tests, and medians using Kruskal-Wallis test. Logistic regression was used to test the associations in a multivariate model using the Wald test.

Medical records of patients diagnosed with HIV infection were reviewed. Data including stage of HIV infection, presence of AIDS defining illness, CD4 count on presentation, as well as links to care, antiretroviral therapy status and outcome were obtained.

This study was reviewed and approved by the Singapore National Health Care Group (NHG) Domain Specific Ethics Review Board Committee.

## Results

During the 24-month review period, TTSH received a total of 93,211 admissions; 51,668 patients were excluded from the HIV screening program based on eligibility criteria. Among those who were eligible, 79% (n = 32,675) opted out of HIV screening. There were a total of 8,868 patients who did not opt out (21% acceptance or uptake rate with 99% of these patients actually receiving an HIV screening test (Table 1).

**Table 1:** Data on Total Admissions and Opt Out Voluntary HIV Testing (January 1, 2009 to December 31, 2010).

**Total Admissions**	**93,211**
**Total number of patients included in HIV screening program**	**41,543 (45%)**
**Total eligible patients who opted out of HIV screening**	**32,675 (35%)** [32,675 / 93,211 total admissions] (78.6%) [32,675/41,543 eligible patients]
**Total eligible patients who opted in to HIV screening**	**8,868 (9.5%)** [8,868 / 93,211 total admissions] (21%) [8,868/41,543 eligible patients]

The profile of the patients included in the screening program is the same as the entire admitted population in TTSH from the same period (2009–2010) in terms of gender, 55% male and 45% female. The age distribution of the patients included in the HIV screening program for the 20–59 year old group is 48% (vs 44% for admitted population in TTSH) and for the 60 and above year old group is 52% (vs 56% for admitted population in TTSH) (Data from Annual report 2009 and 2010, Hospital Statistics, Quality Management Information System, Tan Tock Seng Hospital).

The demographic characteristics of patients who opted in and opted out of the HIV screening program are shown in Table 2. The majority of eligible patients who were tested (63%) were men. The mean age of tested patients was 52 years (range: 21–95 years). Chinese ethnicity account for 61% of those tested, while 17% are Malay and 17% are Indians. In univariate analysis, the opt out rate was significantly higher among females (OR: 1.5, 95% CI 1.4–1.6), among those aged >60 years (OR: 2.3, 95% CI 2.2–2.4) and among Chinese ethnicity (OR: 1.7, 95% CI 1.6–1.8). The association with age persists in a multivariate regression analysis after adjustment on ethnicity and gender.

**Table 2:** Demographic characteristics of patients included in the HIV screening program.

Variables	Opt Inn = 8,868 (%)	Opt Outn = 32,625 (%)	Odds Ratio(95% CI)	p value
**Gender**				
Male	5,576 (24)	17,332 (76)	1	
Female	3,392 (17)	15,343 (82)	1.5 (1.4–1.6)	<0.01
**Age**				
20–59	5,684 (29)	14,200 (71)	1	
60–109	3,184 (15)	18,475 (85)	2.3 (2.2–2.4)	<0.001
Mean (SD)	52.7 (16.6)	60.7 (17.2)	-	<0.001
Median (IQR)	53 (41–65)	62 (49–74)	-	<0,001
**Ethnicity**				
Chinese	5,418 (19)	23,881 (82)	1.7 (1.6–1.8)	<0.001
Indian	1,336 (29)	3,347 (71)	1	
Malay	1,514 (28)	3,824 (72)	1.0 (0.9–1.1)	>0.20
Others	600 (27)	1,623 (73)	1.1 (1.0–1.2)	0.18

Sixty-six patients had a reactive HIV antibody test, of which 15 were confirmed to have HIV infection by a positive Western blot assay; 47 had a negative Western blot. There were 4 indeterminate Western blot results −1 false positive (undetectable HIV RNA assay), 2 did not have further follow up after being advised to undergo repeat HIV testing, and 1 had acute HIV seroconversion illness. The false positive rate of the HIV screening test is 0.56%.

All 16 confirmed HIV patients represented new HIV diagnoses. Table 3 summarizes the demographic and clinical characteristics of these patients. All patients were men and 75% were of Chinese ethnicity. The median age was 51 years (range 23–74 years) with 3 patients >70 years old. The median CD4 count was 150 cells/mm^3^ (range: 20–543 cells/mm^3^) with 69% having a CD4≤200 cells/mm^3^. Five patients presented with an AIDS-defining condition. All 16 patients were referred to an infectious disease (ID) specialist. The median duration to being seen by an ID specialist from the date of referral was 8 days (range: 0 to 20 days). At time of review of medical records, 11 patients remain in active follow up; 9 are receiving antiretroviral (ARV) therapy. The 2 patients not receiving treatment do not require ARV based on current HIV treatment guidelines. Among the 5 patients who are not in active follow up, 1 has died, 1 is receiving palliative care for a non AIDS defining malignancy and 3 have not followed up in clinic as scheduled or have defaulted. The reasons for defaulting follow up included not being ready to accept the HIV diagnosis and refusal to take antiretroviral therapy.

**Table 3:** Characteristics of HIV Patients diagnosed through Screening Program.

	n
**Number of HIV patients**	16
**Sex:**	
**- Male**	16
**Median Age (range)**	51 y (23–75)
**Ethnicity:**	
**- Chinese**	12
**- Malay**	3
**- Indian**	1
**Risk Factors:**	
**- MSM**	3
**- Bisexual**	1
**- Heterosexual**	11
**- Unknown**	1
**Previous HIV Testing:**	
**- No previous HIV Test**	12
**- Previous HIV Test**	2
**- Unknown HIV testing history**	2
**Presence of AIDS-Defining Illness on diagnosis**	5
**Median CD4 count (range)**	150 (<20 to 543)
**Other medical comorbidities** ∧	18
**Median number of days from date of HIV test to referral to ID (min-max)**	8 days (1 to 300)
**Median number of days from date of referral to being seen by ID Specialist**	8 days (0 to 20)
**Outcome:**	
**- In active follow up:**	11
**Receiving antiretroviral therapy**	9
**Not receiving antiretroviral therapy**	2∧∧
**- Default**	3
**- Palliative Care**	1
**- Death**	1
**Number of patients in active follow up receiving antiretroviral therapy**	9

∧ Medical comorbidities include: hypertension, diabetes, hyperlipidemia, coronary artery disease, depression, gout, benign prostatic hyperplasia, chronic hepatitis B, alcoholic liver disease.

∧∧ Not indicated to receive antiretroviral therapy based on current WHO HIV treatment guidelines.

Table 4 shows the commonly cited reasons for refusing an HIV test. The most common reason patients reported for opting out was that patients did not think that they had risk factors for acquiring HIV infection, followed by fear of venipuncture.

**Table 4:** Reasons cited for opting out of HIV Screening Test.

Reason	N	%
**Financial Issue**	24	9.3
**Deemed no risk factors (ie, old age, no sexual contact)**	144	55.8
**Fear of blood test/needles**	58	22.4
**Others** *	32	12.4
**Total**	258	100.0

* **Other reasons cited include: fear of results, family objected, wants to resolve current medical illness first, deemed low risk.**

## Discussion

While the prevalence of HIV in Singapore remains low compared to other countries in Southeast Asia and globally, the majority of newly diagnosed HIV individuals are in the advanced stage of the infection. Patients are most often identified as HIV infected when they present with clinical symptoms or with an AIDS-defining condition [1]. The delay from HIV infection to testing and treatment represents a lost opportunity for early HIV treatment, as well as, prevention of HIV transmission risk to uninfected partners.

Our program showed that 55% of all patients admitted to our hospital are excluded from the opt out HIV testing program based on exclusion criteria. And among those who were eligible, only 21% agreed to the HIV test. All patients diagnosed with HIV were linked to care.

### Low Proportion of HIV among those tested:

The proportion of patients who were positive among those who tested was low at 0.18%. Compared with other testing strategies (Table 5), the lower prevalence can be due to the reason that those who agree to testing have lower pre test probability of having HIV infection. In comparison, in 2007, the anonymous testing site of Action for AIDS in Singapore reported an overall prevalence rate of 1.8%, with the majority (5.7%) accounted for by men having sex with men (MSM) while heterosexual males accounted for 0.8% [7].

**Table 5:** Comparison of various HIV screening tests conducted in Singapore.

3 HIV Screening Strategies	Number of Individuals Tested	Group Tested	HIV Testing uptake, %	Proportion of HIV among those tested, %
**A**	6,903	High risk	-	1.8%
**B**	8,868	Low to High risk	21%	0.18%
**C**	15,945	Low risk	99.42%	0.05%

A : January 2007 to December 2007: Action For AIDS (AFA)- Anonymous Testing Site^6^ : [MSM-5.7%, Heterosexual male-0.8%, Heterosexual female- 0.4%].

B: January 2009 to December 2010.

TTSH- Routine Voluntary Opt Out Inpatient HIV testing Program (This study).

C: January 2010 to December 2010 to Antenatal Screening From acute public hospitals and polyclinics (Data from Communicable Disease Division, MOH, Singapore 2010).

The high false positive rate we noted in our study (0.56%) could be accounted for by the very low prevalence rate of HIV among those screened.

However, assuming the prevalence of HIV is similar among various inpatients in major hospitals and that HIV infected patients present as inpatients at the same frequency, we estimate from our findings that this testing effort could identify around 40–50 HIV infected patients per year. Therefore the absolute yield from this testing strategy is potentially significant. Studies of routine HIV testing done in the US have been conducted with calculation of the costs of identifying and treating HIV cases measured against the cost of averting potential new infections. Results have shown the cost effectiveness of this strategy [8]–[10].

### High Proportion of patients who are excluded from Opt Out HIV testing based on eligibility criteria

In our study, the voluntary opt out HIV testing strategy in the inpatient setting misses 55% of the patients admitted based on baseline exclusion criteria. The proportion of patients who had an HIV screening test among all patients admitted in the hospital during the period was only 9.5% (Table 1). Screening in the inpatient setting will miss patients who may be too ill to consent to HIV testing. A complementary testing strategy would be to routinely offer HIV testing to patients in the outpatient primary care setting, and package HIV testing with other standard tests such as cholesterol and blood glucose screening.

### Low Acceptance Rate of HIV testing

Our overall opt in rate was low at 21%. The low acceptance rate of HIV testing in Singapore remains a major obstacle in assessing the true yield of this testing strategy. Limited literature on routine inpatient HIV testing varies from 24% acceptance rate in a Veterans Affairs Hospital to 64.9% in an urban hospital with an emergency department using a rapid HIV testing program in the United States [5], [6]. Table 5 shows the higher acceptance rate during antenatal HIV screening. This is not surprising given that mothers do not want to inadvertently pass HIV to their infants.

The most common reason for declining HIV testing was the patient's perception that they were at low risk or had no risk of contracting HIV. Studies from other countries show that there is a significant discrepancy between respondents acknowledging risk behavior and perceiving risk, and that individuals tend to underestimate their potential risk for HIV infection [11].

Our evaluation shows that patients with characteristics including Chinese ethnicity, age ≥60 years are more likely to opt out of HIV testing. This is a concern for a number of reasons. First, according to MOH data, the proportion of newly diagnosed older HIV patients for the past 10 years is between 27–30% [1]. Second, data also shows that compared to younger newly diagnosed HIV individuals, the older HIV individual tend to have a lower CD4 count (Data, HIV Programme, Communicable Disease Centre, 2008–2010). The lower CD4 count among older HIV individuals relates either to the age or the late stage at which HIV is diagnosed. Older individuals as well as their medical providers may not have an adequate perception of HIV risk. Prevention campaigns in Singapore have also generally targeted younger people and among the MSM community. Likewise, HIV testing campaigns have not been geared toward older adults. Without proper perception of individual risks, it is possible that opt out HIV testing will not improve uptake of HIV screening as most of the patients will deem themselves without risk and thus do not see the need of having an HIV test. Increased awareness among doctors is likewise needed so that patients are assessed routinely for risk factors for HIV regardless of age and sexual orientation and are offered HIV testing regularly in the course of medical care.

Venipuncture has been implicated as a barrier to patients receiving an HIV test in our program, suggesting that the availability of saliva-based rapid testing maybe a key to improving patient acceptance. However, the higher cost of the rapid test may present a barrier not present for the government-subsidized serum-based HIV test. The current cost of the oral based test in Singapore is around S$28 compared to the subsidized price of the blood based HIV test (S$6–10). A possible approach would be to perform the HIV test at the same time as another blood test during the hospitalization, to avoid additional venipuncture by collecting a separate dedicated tube for the HIV test.

Finally, fear of receiving a positive HIV test result was mentioned as a reason for opting out. Patients may fear dying from HIV, the financial burden of antiretroviral treatment, and the loss of job, family, and friends. It is evident that the major challenges of HIV in Singapore remains to be HIV related stigma and discrimination [12]. A National Survey on public attitudes towards people living with HIV/AIDS (PLWHA) conducted in 2007 show that 54.1% of respondents would care for a close relative who became ill due to HIV/AIDS in their own home, 22.4% are willing to share a meal with a person living with HIV and only 18.2% would buy food from a shopkeeper living with HIV [13]. Acknowledging this major obstacle, the Ministry of Health (MOH) together with community partners have increased their efforts to address stigma and discrimination towards people living with HIV.

### Limitations

We were only able to survey the reasons for opting out of HIV testing only from a small proportion of patients. There might be possible differences in this group compared to those who were not surveyed. A more extensive survey may be warranted in the future to understand the reasons for opting out. The other limitation of our study is the generalizability of our results to other Singapore hospitals and the general population.

While we have shown that it is possible to conduct routine voluntary opt out inpatient HIV testing in our hospital, and that those that opt in and receive a new HIV diagnosis are successfully linked to care, more efforts are needed to address reasons for opting out of HIV testing. This could include education campaigns that address individual risk perception for HIV in conjunction with anti HIV stigma and anti-discrimination campaigns in the general population.
